# The impact of change in neighborhood poverty on BMI trajectory of 37,544 New York City youth: a longitudinal study

**DOI:** 10.1186/s12889-020-09772-5

**Published:** 2020-11-10

**Authors:** Krista Schroeder, Sophia Day, Kevin Konty, Levent Dumenci, Terri Lipman

**Affiliations:** 1 Temple University College of Public Health, 1101 West Montgomery Avenue, Philadelphia, PA 19122 USA; 2 New York City Department of Health and Mental Hygiene, Office of School Health, 42-09 28th Street, 14th floor, Long Island City, NY 11101 USA; 3 University of Pennsylvania School of Nursing, 418 Curie Boulevard, Philadelphia, PA 19140 USA

**Keywords:** Pediatric obesity, Poverty, Neighborhood

## Abstract

**Background:**

Neighborhood poverty may increase childhood obesity risk. However, evidence for the neighborhood poverty-obesity relationship is limited. The purpose of this study was to examine how moving to a higher or lower poverty neighborhood impacts body mass index (BMI) z-score trajectories among youth, with the goal of informing policy change, interventions, and clinical practices to reduce childhood obesity.

**Methods:**

Methods entailed secondary analysis of existing longitudinal data. The sample included youth attending New York City public schools in grades kindergarten through twelfth from school years 2006/2007 through 2016/2017. Eligibility criteria included moving to a higher or lower poverty neighborhood during the data midpoint [school years 2010/2011 through 2013/2014] of the 12-year data-period; New York City-specific metrics were used to define both neighborhood (Neighborhood Tabulation Area) and relevant neighborhood poverty levels (< 5, 5 to < 10%, 10 to < 20%, 20 to < 30%, 30 to < 40% and ≥ 40% of individuals below Federal Poverty Level). Two-piece latent growth curve models were used to describe BMI z-score trajectories of youth who moved to higher versus lower poverty neighborhoods, with propensity score weighting to account for preexisting differences between the two groups. Primary analyses were stratified by sex and exploratory subgroup analyses were stratified by sex and developmental stage (early childhood, middle childhood, and adolescence) to explore sensitive periods for neighborhood poverty exposure.

**Results:**

Of 532,513 youth with home address data, 18,370 youth moved to a higher poverty neighborhood and 19,174 moved to a lower poverty neighborhood (*n* = 37,544). Females and males who moved to a higher poverty neighborhood experienced less favorable BMI z-score trajectories for obesity risk, though effects were small. Exploratory subgroup analyses demonstrated that negative effects of neighborhood poverty were most pronounced among young and adolescent females and young males, whereas effects were mixed for other subgroups.

**Conclusions:**

Youth who moved to higher poverty neighborhoods experienced less favorable BMI z-score trajectories for obesity risk, though effects were small and most consistent for females and younger youth. Additional research is needed to illuminate neighborhood poverty’s impact on obesity, in order to inform policy, intervention, clinical, and research efforts to reduce obesity and improve child well-being.

**Supplementary Information:**

The online version contains supplementary material available at 10.1186/s12889-020-09772-5.

## Background

Pediatric obesity impacts 6–8% of youth worldwide [1] and 18.5% of youth in the United States [2], increasing their risk for excess school absences, poorer academic performance, greater use of medical service, low self-esteem, worse health-related quality of life, and multiple chronic diseases [3–13]. Obesity prevention efforts from the past two decades have had limited impact [14]. Traditional obesity interventions targeting energy balance and individual health behavior change (e.g., caloric restriction, increased exercise) have not substantially decreased obesity prevalence [15, 16]. Causes of obesity are not fully understood, despite extensive research. Genetic, physiological, individual, and environmental factors contribute to obesity, and no single factor is sufficient to explain the obesity crisis. In particular, a narrative of personal responsibility and a sole focus on health behavior change is no longer advocated [17, 18]. Recent efforts call for more deeply examining and teasing apart distal factors that contribute to obesity to inform policy, interventions, and clinical care [19–23].

Research examining how neighborhood impacts obesity is particularly needed, in order to drive public and political support for evidence-based environmental policy change [24]. Various neighborhood characteristics may contribute to obesity including availability of green space, access to supermarkets, pollution, traffic, social norms, and safety/crime [25–29]. However, it is difficult to disentangle the influence of each neighborhood characteristic because many overlap with one another and occur within the broader context of neighborhood racial/ethnic/economic segregation. Further, neighborhood impact on health is complex, being influenced by culture, level of social cohesion, and sociopolitical structures; contextual factors that are difficult to capture (such as social norms and beliefs) vary across neighborhoods and influence health outcomes [30, 31]. Various approaches to understanding neighborhood influence exist. Some studies examine neighborhood characteristics collectively via summary measures such as the neighborhood deprivation index [32] - an approach well suited to understanding how overall neighborhood environment collectively impacts obesity; in contrast, other studies examine an individual neighborhood characteristic (such as access to supermarkets) in isolation - an approach well suited to informing focused policy change [33].

Poverty is a key neighborhood characteristic that has recently received increased attention in efforts to establish the relationship between neighborhood and obesity [24, 34–36]. Neighborhood poverty may impact obesity via two potential pathways [37]. The first and most commonly recognized pathway is via unhealthy neighborhood conditions. Low-income neighborhoods, especially in urban areas, have experienced historical disinvestment and are less likely to have health promoting resources such as grocery stores selling fresh produce, bike share programs, and well-lit and maintained parks, sidewalks, and trails. Clinical care, including specialty care such as pediatric obesity clinics and nutritionists, are often absent. Pollution from traffic and manufacturing is more common in low-income neighborhoods and may contribute to obesity via inflammation, decreased physical activity, and increased chronic disease risk [38]. The second pathway linking neighborhood poverty and obesity is related to stress. Living in a high poverty neighborhood, with its associated higher rates of crime, limited access to resources, and associated internal and external stigma can lead to toxic stress - a chronic activation of the stress system without access to appropriate buffering resources [39–41]. Chronic stress, and resulting neuroendocrine responses such as elevated cortisol, can increase abdominal adiposity storage, spur cravings for highly palatable calories-dense foods, and disrupt sleep needed to support a healthy body weight [42].

Despite hypothesized underpinnings, the neighborhood poverty-obesity relationship is not well understood [37, 43]. Research on this relationship is challenging because randomizing youth to higher or lower poverty neighborhoods is unethical, making randomized controlled trials unfeasible. In addition, effects of neighborhood poverty are likely not immediate and thus long-term data collection is required. Further, there are often key differences between children who live in high versus low poverty neighborhoods; for example, members of racial/ethnic minority groups are more likely to live in high poverty neighborhoods regardless of family income due to long-standing structural inequities and racism [44]. High quality studies examining the impact of neighborhood poverty on obesity are needed to inform policy and identify populations for targeted interventions and clinical care [43].

### Purpose

The purpose of this paper was to examine how moving to a higher or lower poverty neighborhood impacts youths’ body mass index (BMI) z-score trajectories. Specifically, we aim to describe BMI z-score trajectories of two groups: youth who moved to a higher poverty neighborhood and youth who moved to a lower poverty neighborhood. The final sample included 37,544 youth attending New York City public schools from 2006 to 2017, with propensity score weighting used to account for preexisting differences between youth who moved to higher (*n* = 18,370) versus lower (*n* = 19,174) poverty neighborhoods. We hypothesized that youth who moved to a higher poverty neighborhood would demonstrate increase in BMI z-score, with the opposite effect for youth who moved to a lower poverty neighborhood. The overarching goal was to contribute to a better understanding of how poverty impacts obesity and inform policies, interventions, and clinical practices for obesity reduction in high poverty neighborhoods.

## Methods

### Data sources

This study entailed a secondary analysis of existing data. The primary data source was New York City (NYC) Fitnessgram. The NYC Fitnessgram program entails annual collection of height and weight of all children attending NYC public schools in grades kindergarten through twelve by trained physical education teachers [45]. NYC Fitnessgram data were linked to school administrative data that included student demographic information and geocoded home address for each school year. Each year of data from school year 2006/2007 (the first year of NYC Fitnessgram) through 2016/2017 was used in this study; years were linked to each other and the school administrative data by a unique student identifier. The neighborhood poverty data source was the 2015 Census American Community Survey [46], which was linked to the NYC Fitnessgram data by Census tract of the student’s home address.

### Measures

Demographic data included student sex (male/female), race/ethnicity (Asian Pacific Islander/Black non-Hispanic/White non-Hispanic, Native American or Alaskan Indian, Hispanic), age in years (continuous), grade (kindergarten through twelfth), eligibility for free/reduced lunch (yes/no), and English language learner status (yes/no). BMI z-score was calculated from height, weight, and sex per standard procedures using Centers for Disease Control and Prevention growth charts [47]; BMI z-score data and calculation methods for youth attending NYC public schools have been published elsewhere [48–50].

Neighborhood was defined as Neighborhood Tabulation Area. Neighborhood Tabulation Areas are NYC-specific aggregations of Census tracts that reflect neighborhoods. Neighborhood Tabulation Areas provide a more statistically reliable measure of neighborhoods than Census tracts, given the high level of sampling error in American Community Survey Census tract data [51]. In addition, Neighborhood Tabulation Areas reflect commonly defined neighborhoods (e.g., ‘Upper East Side’) rather than solely administrative boundaries. Neighborhood poverty was defined as percent of individuals below the Federal Poverty Level using five year estimates from the American Community Survey [46]. Poverty was divided into six categories (< 5, 5 to < 10%, 10 to < 20%, 20 to < 30%, 30 to < 40% and ≥ 40% below Federal Poverty Level), using categories identified by the Public Health Disparities Geocoding Project and tailored for NYC [52]. Thus, to summarize the above, moving to a higher or lower poverty neighborhood poverty was operationalized as moving to a Neighborhood Tabulation Area with a higher or lower poverty category.

### Sample

NYC Fitnessgram data included youth attending NYC public schools from kindergarten through twelfth grade from school year 2006/2007 through school year 2016/2017 [49, 53, 54], of which 532,513 had home address data. Eligibility criteria for this study included a) moving to a higher or lower poverty neighborhood at the approximate midpoint [school year 2010/2011 through 2013/2014] of the twelve year data period and b) not missing demographic data required for propensity score application [described below]. Defining the move period as the data midpoint allowed for examining the BMI z-score trend over time - specifically both before and after the move. To ensure the cleanest possible comparison, youth who moved to higher or lower poverty neighborhood multiple times during the midpoint, never moved, or moved before/after the midpoint were excluded. From the eligible population, two groups were created: youth who moved to a higher poverty neighborhood, and youth who moved to a lower poverty neighborhood.

### Application of propensity score

Propensity score methods are commonly used to address bias in observational studies where the exposure of interest cannot be or was not randomly assigned [55–57]. While propensity scores cannot mimic randomization, the method may be superior to traditional methods such as matching or multivariate regression in accounting for confounder effects [55, 58, 59]. In this study, propensity scores were used to account for differences between youth who moved to a higher poverty neighborhood and youth who moved to a lower poverty neighborhood. Sex, race/ethnicity, age and grade at time of move, BMI and BMI z-score at time of move, race/ethnicity, grade level at time of move, eligibility for free/reduced lunch, and English language learner status were included in the propensity score [60]. Propensity scores were created and fit assessed using standard methods [57, 61, 62]. Two propensity score methods were compared (propensity score matching and propensity score weighting) to determine which best reduced confounder imbalance for this sample. Consistent with other studies comparing propensity score methods [63–68], propensity score weighting was more effective than matching for this sample, though differences were not substantial. Specifically, propensity score weighting led to fewer significant differences between groups (2 differences for weighting versus 4 differences for matching) and the remaining significant differences were not clinically meaningful (i.e., age in months differed by 0.01, BMI differed by less than 0.01). Thus, propensity score weighting was used for data analysis [56].

### Data analysis

Demographic characteristics were assessed using standard descriptive statistics, with measures of central tendency and variation used for continuous variables and frequencies and percentages for categorical variables. Multi-group (higher versus lower poverty neighborhood) two-piece (before and after the move) latent growth curve analysis was used to estimate stability and change in BMI z-score over a 12-year period. The 12-year period was divided into a before and after the move trajectory, based on each child’s year of moving. For example, a youth who moved in 2012 would have six years of pre-move BMI data (school years 2006/2007–2011/2012) and six years of post-move BMI data (school years 2012/2013–2017/2018), and a youth who moved in 2011 would have five years of pre-move BMI data (school years 2006/2007–2010/2011) and seven years of post-move BMI (school years 2011/2012–2017/2018). Given variation in participant age, not all participants had BMI data for the full 12-year period (e.g., a tenth grade student who moved in 2012/2013 would graduate before 2016/2017); missing BMI data was handled via full information maximum likelihood as described below. The latent growth factors included a common intercept plus linear and quadratic changes over time before and after the move. Multi-group analysis allowed for estimating changes in BMI z-score for the higher and lower poverty neighborhoods simultaneously while also allowing for between-group differences in means and variances of growth factors. “Two-piece” latent growth curve analysis was chosen given the aim of examining similarities and differences in BMI z-score trajectories after the potentially moving to a higher or lower poverty neighborhood. (Note: Growth curves of youth who didn’t move, and thus were excluded from the current study, were examined as a posthoc exploratory analysis; their trajectories, by poverty category, and are presented in Additional File 1).

Primary analyses were stratified by sex, given hypothesized differences in response to change in neighborhood poverty [69, 70]. Secondary analyses were stratified by sex and developmental stage (young childhood < 10 years, middle childhood 10 to < 13 years, and adolescent ≥13 years) given differences in BMI z-score trajectory as children age (e.g., adiposity rebound, impact of puberty and growth) and to explore sensitive periods for poverty exposure [71]. All analyses were weighted by propensity score. A robust maximum likelihood method was used to take account of non-normal outcome distributions (BMI z-score) and the full information maximum likelihood to handle missingness by using all available data during model estimation. Analyses were conducted using SAS (propensity scores and descriptive statistics) [72] and Mplus (latent growth curve analysis) [73].

## Results

Sample demographics are in Table 1. Of the 532,513 youth with address data in the NYC school system from 2006/2007 through 2016/2017, 488,732 did not move to a higher or lower poverty neighborhood during the move period and 6137 were missing data on demographics required for propensity score matching, resulting in 37,544 youth who met eligibility criteria. Of those, 18,370 moved to higher poverty neighborhoods and 19,174 moved to lower poverty neighborhoods. Approximately half were female (49.6%). Mean age was 10.9 ± 3.0 years and 17,043 (45.5%) were in young (< 10 years) childhood, 11,502 (30.64%) in middle (10 to < 13 years) childhood, and 8998 (23.97%) in adolescence (≥13 years). Mean BMI z-score was 0.62 ± 1.18 and 15.8% were English language learners. Regarding race/ethnicity, 10.02% were non-Hispanic White, 0.42% were Native American or Alaskan Indian, 42.41% were Hispanic, 34.11% were non-Hispanic Black, and 12.84% were Asian or Pacific Islander. Roughly one-fifth (21.03%) met criteria for obesity.

**Table 1:** Demographics at time of moving to a higher or lower poverty neighborhood, for youth attending New York City schools from 2006/2007 through 2016/2017

Characteristic	Total Sample (*n* = 37,544)	Moved to Higher PovertyNeighborhood (***n*** = 18,370)	Moved to Lower PovertyNeighborhood (***n*** = 19,174)	***P*** value for difference
Age in years (mean [SD])	10.42 ± 2.98	10.42 ± 3.02	10.42 ± 2.95	< 0.01
Sex (n [percent])				0.99
Male	18,929 (50.42)	9463 (50.42)	9466 (50.42)	
Female	18,615 (49.58)	9306 (49.58)	9310 (49.58)	
Race/ethnicity (n [percent])				0.99
Asian/Pacific Islander	4822 (12.84)	2408 (12.83)	2414 (12.86)	
Non-Hispanic black	12,806 (34.11)	6403 (34.11)	6403 (34.10)	
Hispanic	15,922 (42.41)	7961 (42.42)	7961 (42.40)	
Native American/Alaskan Indian	158 (0.42)	79 (0.42)	79 (0.42)	
Non-Hispanic white	3762 (10.02)	1881 (10.02)	1881 (10.02)	
Other (missing/multiracial)	75 (0.20)	37 (0.20)	37 (0.20)	
Body mass index (mean [SD])	20.05 ± 4.97	20.05 ± 5.01	20.05 ± 4.93	< 0.01
Body mass Index z-Score (mean [SD])	0.62 ± 1.18	0.62 ± 1.19	0.62 ± 1.61	0.48
Free/reduced school meals (n [percent])				0.99
No	2892 (7.70)	1445 (7.70)	1446 (7.70)	
Yes	34,653 (92.30)	17,324 (92.30)	17,329 (92.30)	
English language learner (n [percent])				0.97
No	31,611 (84.20)	15,804 (84.20)	15,807 (84.19)	
Yes	5933 (15.80)	2965 (15.80)	2969 (15.81)	
Obesity				< 0.01
No	29,649 (78.97)	14,843 (79.09)	14,805 (78.86)	
Yes	7895 (21.03)	3925 (20.91)	3970 (21.14)	

**Fig. 1:**
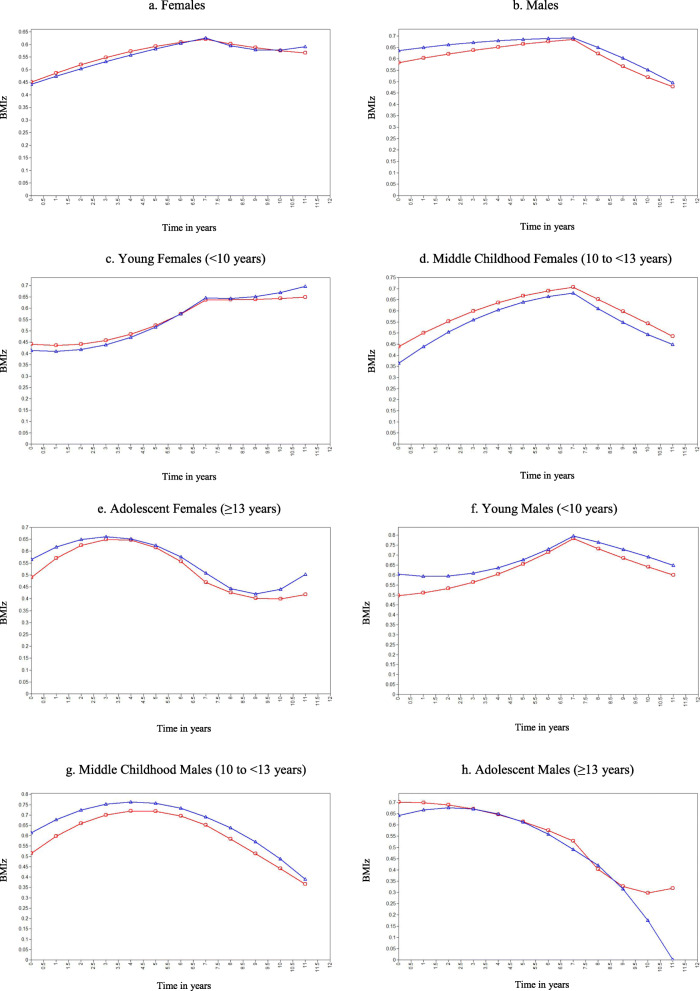
BMI z-score trajectory after moving to higher versus lower poverty neighborhood for youth attending New York City schools from 2006/2007 through 2016/2017. Note: Red = moved to lower poverty neighborhood, blue = moved to higher poverty neighborhood

Models fitting pre- and post-move BMI z-score trajectories are shown in Fig. 1 and associated parameters are shown in Table 2. Trajectories include both linear slope (indicating BMI z-score linear change) and a quadratic term (indicating BMI z-score change acceleration). (Note: Additional File 2 shows the trajectories with 95% confidence intervals.)

**Table 2:** Results of two part latent growth curve modelling demonstrating BMI z-score trajectory before and after moving to a higher or lower poverty neighborhood, for youth attending New York City schools from 2006/2007 through 2016/2017

Parameter	Moved to Higher Poverty Neighborhood				Moved to Lower Poverty Neighborhood			
	Before Move		After Move		Before Move		After Move	
	Mean	Variance	Mean	Variance	Mean	Variance	Mean	Variance
**FEMALES**								
BMIz at time of move (intercept)	0.626***	1.092***			0.621***	1.089***		
BMIz change (linear slope)	0.020***	0.076***	−0.038***	0.129***	0.010*	0.076***	−0.020**	0.127***
BMIz acceleration (quadratic slope)	− 0.001^ns^	0.001***	0.007***	0.007***	−0.002**	0.001***	0.002^ns^	0.007***
**Young females (< 10 years)**								
BMIz at time of move (intercept)	0.646***	1.196***			0.635***	1.163***		
BMIz change (linear slope)	0.077***	0.076***	−0.007^ns^	0.132***	0.067***	0.078***	0.001^ns^	0.133***
BMIz acceleration (quadratic slope)	0.006***	0.002***	0.005*	0.009***	0.006***	0.002***	0.001^ns^	0.007***
**Middle childhood females (10 to < 13 years)**								
BMIz at time of move (intercept)	0.680***	1.001***			0.706***	0.977***		
BMIz change (linear slope)	0.011^ns^	0.066***	−0.074***	0.093***	0.012 ^ns^	0.069***	−0.053***	0.115***
BMIz acceleration (quadratic slope)	−0.005***	0.001***	0.004^ns^	0.004***	−0.004**	0.001***	0.000^ns^	0.006***
**Adolescent females (≥13 years)**								
BMIz at time of move (intercept)	0.507***	0.996***			0.469***	1.062***		
BMIz change (linear slope)	−0.078***	0.079***	−0.086***	0.194**	−0.101***	0.067***	−0.053*	0.207***
BMIz acceleration (quadratic slope)	−0.010***	0.002***	0.021*	0.018*	−0.014***	0.001***	0.010^ns^	0.022**
**MALES**								
BMIz at time of move (intercept)	0.692***	1.174***			0.684***	1.182***		
BMIz change (linear slope)	0.001^ns^	0.092***	−0.039***	0.157***	0.008^ns^	0.084***	−0.067***	0.146***
BMIz acceleration (quadratic slope)	−0.001^ns^	0.002***	−0.003^ns^	0.008***	−0.001^ns^	0.001***	0.004^ns^	0.008***
**Young males (< 10 years)**								
BMIz at time of move (intercept)	0.797***	1.154***			0.783***	1.160***		
BMIz change (linear slope)	0.073***	0.083***	−0.031**	0.131***	0.073***	0.089***	−0.054***	0.141***
BMIz acceleration (quadratic slope)	0.007***	0.002***	−0.002^ns^	0.007***	0.005**	0.002***	0.002^ns^	0.008***
**Middle childhood males (10 to < 13 years)**								
BMIz at time of move (intercept)	0.691***	1.212***			0.651***	1.198***		
BMIz change (linear slope)	−0.050***	0.092***	−0.045***	0.198***	−0.054***	0.074***	−0.066***	0.145***
BMIz acceleration (quadratic slope)	−0.009***	0.002***	−0.008*	0.010***	−0.011***	0.001***	−0.001^ns^	0.007***
**Adolescent males (≥13 years)**								
BMIz at time of move (intercept)	0.491***	1.102***			0.529***	1.156***		
BMIz change (linear slope)	−0.076***	0.091***	−0.053**	0.118***	−0.050***	0.077***	−0.150***	0.200**
BMIz acceleration (quadratic slope)	−0.008***	0.002***	−0.017*	0.003^ns^	−0.004*	0.001***	0.024*	0.014^ns^

Note. *p < 0.05; **p < 0.01; ***p < 0.001, ns = not statistically significant at p < 0.05 cutoff; BMIz = Body mass index z-score; The mean and variance parameters of the intercept are the same before and after move

Females who moved to a higher poverty neighborhood experienced a greater BMI z-score linear decrease but also a greater BMI z-score acceleration compared to those who moved to a lower poverty neighborhood, resulting in a higher final post-move BMI z-score (Fig. 1a). Males who moved to a higher poverty neighborhood experienced a smaller BMI z-score linear decrease than those who moved to a lower poverty neighborhood; however overall trajectories and final post-move BMI z-score were similar (Fig. 1b).

Subgroup analysis illuminated differences by age group. Females who moved as young children (< 10 years) to a higher poverty neighborhood had a greater BMI z-score acceleration than those who moved to a lower poverty neighborhood, resulting in a higher final post-move BMI z-score (Fig. 1c), as did females who moved as adolescents (≥13 years) (Fig. 1e). In contrast and contrary to our hypothesis, females who moved to a higher poverty neighborhood during middle childhood (10 to < 13 years) experienced a greater BMI z-score linear decrease than those who moved to a lower poverty neighborhood; however overall trajectories were similar (Fig. 1d). Males who moved as young children (< 10 years) to a higher poverty neighborhood experienced a smaller BMI z-score linear decrease than those who moved to a lower poverty neighborhood, resulting in a higher final post-move BMI z-score (Fig. 1f). Boys who moved during middle childhood (10 to < 13 years) demonstrated similar trajectories among those who moved to higher and lower poverty neighborhoods, with a similar final post-move BMI z-score (Fig. 1g). Males who moved as adolescent (≥13 years) also experienced similar trajectories for both groups, though males who moved to lower poverty neighborhoods experienced a decrease in BMI z-score at the end of the post-move period (Fig. 1h).

## Discussion

Our study examined the impact of moving to a higher versus lower poverty neighborhood for 36,544 youth attending NYC public schools in kindergarten through twelfth grade. Results demonstrated that youth who moved to higher poverty neighborhoods experienced less favorable BMI z-score trajectories for obesity risk, though effects were small and most consistent for the youngest youth. Our findings suggest that the impact of neighborhood poverty on obesity may be most salient before pre-adolescence, and are consistent with other literature indicating the key impact of obesity risk factors during early years of development [74, 75]. Further, our findings are consistent with the anecdotal observation that younger youth spend more time with parents/caregivers and thus in close proximity to their home neighborhood - whereas older youth are more likely to spend time with peers (e.g., at a friend’s house in another neighborhood) or independently exploring other areas (e.g., at a park near their school’s neighborhood school with classmates). Thus, younger youth may be more influenced by their home neighborhood simply because they spend more time there.

Our study contributes to the complex literature about poverty’s effect on obesity. Potential contextual (unhealthy conditions) and physiological (stress) pathways exist, but much prior work has focused on family poverty (which is related to but different than neighborhood poverty) [37, 43]. Despite previous research [43] and seminal studies (e.g., Moving-to-Opportunity [70]), neighborhood poverty’s effects remain unclear [43]. Future research is needed to examine neighborhood poverty’s association with obesity, as well as associations with obesity risk factors (e.g., low fitness, poor nutrition) [76–78]. Given that randomization to neighborhoods is not ethical nor feasible, strong observational studies remain necessary to deconstruct the impact of this important social determinant of health on obesity risk. While there are many ethical arguments for addressing poverty, studies that build the quantitative evidence base for its eradication can add to the case.

The relationship between neighborhood factors, such as poverty, and obesity are particularly important to local governments and health departments [79–81]. Local government and health department action has the ability to directly impact neighborhood environment through actions related to zoning, parks, and provision of services [79–81]. Further, a larger local government like New York City - the setting for this study - represents a population with tremendous diversity in characteristics (neighborhoods differ) and outcomes (neighborhoods demonstrate disparities) [29, 82–85]. Thus, understanding the link between neighborhood and health is critically important to understanding and addressing local health outcomes, such as obesity.

Obesity, as with many complex chronic conditions, doesn’t have a sole cause. Unlike causal agents related to communicable disease risk, causal agents for non-communicable diseases like obesity are likely to be modest, incremental, and synergistic. Research can identify and tease apart relevant factors, but it would be unrealistic to expect any single factor to have an enormous and direct impact. For example, studies examining neighborhood characteristics such as the introduction of supermarkets to food deserts have demonstrated modest or mixed effects [26, 33]; however this does not mean that adding supermarkets to food deserts is futile - it simply demonstrates that supermarkets are only one contributor to multifaceted obesity risk. Aside from bariatric surgery and pharmacologic agents, most isolated obesity interventions (especially those at higher levels of the ecological model [86, 87]) are less likely to have substantial and lasting impact on obesity but they are nonetheless important and may contribute to incremental reduction in risk.

While this study examined the physical environment (neighborhood), the social environment must also be considered. Often environmental effects on obesity are considered related only to physical factors such as access to green space or locations selling fresh produce. However, a key component of a youth’s environment is social (e.g., culture, norms, and identity of the groups to which they belong). Even if a youth’s physical environment changes (e.g., by moving to a lower poverty neighborhood), the youth’s social environment including their family, friends, faith-based community, and cultural group(s) may remain the same. Future studies can attempt to tease apart how physical and social environments interrelate, as relevant to neighborhood poverty and obesity, as well as what BMI z-score trajectories are the norm within those differing environments.

Of note, our study did not account for reasons for moving to a higher or lower poverty neighborhood. Various family situations may lead to relocation, with different situations impacting BMI z-score trajectory differently. For example, a youth may move to a higher poverty neighborhood because of financial challenges associated with parental separation, death, mental illness, or incarceration; such traumatic experiences increase obesity risk [88–93]. In contrast, a youth may move to a lower poverty, objectively “healthier” neighborhood but experience isolation and stress due to being removed from a physical and social environment in which they felt a sense of membership [94]; such stress could negatively impact youth’s obesity risk. Thus, reason for and potential effects of relocation are complex and additional research is needed. Qualitative studies would be well-suited to illuminating the complex social determinants (e.g., social norms, culture, beliefs) that underlie how change in neighborhood poverty influences obesity-related behaviors [30, 31]. In addition, future quantitative studies can explore whether BMI z-score trajectories differ based on reason for relocation.

Strengths of this study include 12 years of longitudinal data with racially/ethnically and socioeconomically diverse sample, use of propensity scores to minimize confounder imbalance, and large sample allowing for an innovative study design. Weaknesses include lack of generalizability due to including an urban sample, use of a fixed poverty measure that does not reflect neighborhood change such as gentrification, and use of data that were not collected for research purposes.

## Conclusions

Childhood obesity negatively impacts the health of millions of youth, highlighting a need for a better understanding of obesity’s complex risk factors. Neighborhood poverty is one such risk factor that merits closer examination. Our study demonstrated that moving to a higher poverty neighborhood, as compared to a lower poverty neighborhood, may negatively impact obesity risk among youth. Risks, while small, may be particularly salient for younger youth and for females. Collectively, our findings highlight the need for high quality research examining neighborhood policy’s impact on obesity, in order to inform evidence-based policy change, interventions, and clinical practices that decrease obesity risk and improve child well-being.

## Supplementary Information

**Additional file 1.** BMI z-score trajectory, by poverty category, for youth who did not move to a higher or lower poverty neighborhood and attended New York City schools from 2006/2007 through 2016/2017.

**Additional file 2.** BMI z-score trajectory, with 95% confidence interval, after moving to higher versus lower poverty neighborhood for youth attending New York City schools from 2006/2007 through 2016/2017.

## Data Availability

The data that support the findings of this study are available from the New York City Department of Health and Mental Hygiene, but restrictions apply to the availability of the data and the data are not publicly available due presence of personally identifiable information on minors under 18 years of age.

## Abbreviations

BMI: Body mass index
NYC: New York City
