# Supplementary material for: The impact of change in neighborhood poverty on BMI trajectory of 37,544 New York City youth: a longitudinal study
Source: BMC Public Health. 2020 Nov 10;20:1676. doi: 10.1186/s12889-020-09772-5 (PMC7653753; doi:10.1186/s12889-020-09772-5)
Supplement: Supplementary file 1 — Additional file 1. BMI z-score trajectory, by poverty category, for youth who did not move to a higher or lower poverty neighborhood and attended New York City schools from 2006/2007 through 2016/2017. [file 12889_2020_9772_MOESM1_ESM.docx]

Additional File 1. BMI z-score trajectory, by poverty category, for youth who did not move to a higher or lower poverty neighborhood and attended New York City schools from 2006/2007 through 2016/2017. *Note: Poverty category was defined as percent of individuals below Federal Poverty Level, neighborhood was defined as Neighborhood Tabulation Area.*
