# Supplementary material for: The impact of change in neighborhood poverty on BMI trajectory of 37,544 New York City youth: a longitudinal study
Source: BMC Public Health. 2020 Nov 10;20:1676. doi: 10.1186/s12889-020-09772-5 (PMC7653753; doi:10.1186/s12889-020-09772-5)
Supplement: Supplementary file 2 — Additional file 2. BMI z-score trajectory, with 95% confidence interval, after moving to higher versus lower poverty neighborhood for youth attending New York City schools from 2006/2007 through 2016/2017. [file 12889_2020_9772_MOESM2_ESM.docx]

Additional File 2. BMI z-score trajectory, with 95% confidence interval, after moving to higher versus lower poverty neighborhood for youth attending New York City schools from 2006/2007 through 2016/2017. *Note: Red=moved to lower poverty neighborhood, blue=moved to higher poverty neighborhood.*
